# Enhanced production of L-sorbose from D-sorbitol by improving the mRNA abundance of sorbitol dehydrogenase in *Gluconobacter oxydans* WSH-003

**DOI:** 10.1186/s12934-014-0146-8

**Published:** 2014-10-18

**Authors:** Sha Xu, Xiaobei Wang, Guocheng Du, Jingwen Zhou, Jian Chen

**Affiliations:** School of Biotechnology and Key Laboratory of Industrial Biotechnology, Ministry of Education, Jiangnan University, 1800 Lihu Road, Wuxi, Jiangsu 214122 China; Synergetic Innovation Center of Food Safety and Nutrition, 1800 Lihu Road, Wuxi, Jiangsu 214122 China

**Keywords:** L-ascorbic acid, D-sorbitol dehydrogenase, Poly(A/T) tails, Immobilization, Fed-batch fermentation

## Abstract

**Background:**

Production of L-sorbose from D-sorbitol by *Gluconobacter oxydans* is the first step to produce L-ascorbic acid on industrial scale. The *sldhAB* gene, which encodes the sorbitol dehydrogenase (SLDH), was overexpressed in an industrial strain *G. oxydans* WSH-003 with a strong promoter, *P*_*tufB*_. To enhance the mRNA abundance, a series of artificial poly(A/T) tails were added to the 3′-terminal of *sldhAB* gene. Besides, their role in *sldhAB* overexpression and their subsequent effects on L-sorbose production were investigated.

**Results:**

The mRNA abundance of the *sldhAB* gene could be enhanced in *G. oxydans* by suitable poly(A/T) tails. By self-overexpressing the *sldhAB* gene in *G. oxydans* WSH-003 with an optimal poly(A/T) tail under the constitutive promoter *P*_*tufB*_, the titer and the productivity of L-sorbose were enhanced by 36.3% and 25.0%, respectively, in a 1-L fermenter. Immobilization of *G. oxydans*-sldhAB6 cells further improved the L-sorbose titer by 33.7% after 20 days of semi-continuous fed-batch fermentation.

**Conclusions:**

The artificial poly(A/T) tails could significantly enhance the mRNA abundance of the *sldhAB*. Immobilized *G. oxydans*-sldhAB6 cells could further enlarge the positive effect caused by enhanced mRNA abundance of the *sldhAB*.

## Background

L-sorbose is an important carbohydrate that is predominantly used as a starting material in the biosynthesis of L-ascorbic acid [1,2]. The most prominent industrial method of producing L-sorbose is the biotransformation of D-sorbitol to L-sorbose in *Gluconobacter* species or *Acetobacter* species [3,4]. During the process, two hydrogen atoms were removed from one molecular of D-sorbitol to form one molecular of L-sorbose. *Gluconobacter oxydans* is widely used in biotransformation due to its ability to incompletely oxidize D-sorbitol, glycerol, and glucose to L-sorbose [5], dihydroxypropanone [6], and gluconic acid [7], respectively. These oxidation reactions are performed by membrane-bound dehydrogenases located on the outer surface of the cytoplasmic membrane, whereas the oxidation products accumulate in the culture medium.

*G. oxydans* WSH-003 and its relative derivative strains are commonly used for the industrial production of L-sorbose, which is the first step in the production of L-ascorbic acid via the two-step fermentation process [8]. The most significant feature of *G. oxydans* WSH-003 is its ability to produce a high titer of L-sorbose. D-sorbitol is oxidized in the periplasm in a chemo-, regio-, and stereoselective manner to L-sorbose by membrane-bound dehydrogenases [9]. The gene clusters encoding D-sorbitol dehydrogenase (*sldhAB*, 2,670 bp) have been identified from the draft genome sequence of *G. oxydans* WSH-003 [8]. For industrial production, it is preferable to engineer *G. oxydans* cells to further enhance their catalytic properties during the biotransformation processes of D-sorbitol [6,10,11]. For example, by overexpressing a membrane-bound glycerol dehydrogenase gene in *G. oxydans* DSM 2343, the titer of dihydroxyacetone from glycerol was enhanced by 75% [10]. This indicated that an engineered *G. oxydans* strain that overexpresses the *sldhAB* gene could enhance the L-sorbose production.

To overexpress an enzyme, the stability of the corresponding mRNA of the enzyme plays an important role in the process. Polyadenylation is the process to add a poly(A) tail on the 3′-terminal of a RNA [12]. The poly(A) tail consists of multiple adenosine monophosphates and is a stretch of RNA that has only adenine bases. In eukaryotes, the poly(A) tail could be shortened over time. Once the poly(A) tail is short enough, the mRNA could be enzymatically degraded [13]. In some of eukaryotes, the mRNA with short poly(A) tails could be reactivated by re-polyadenylation in the cytosol [12]. In *E. coli* mRNA with poly(A) tail could be formed by the activity of poly(A) polymerase [14]. Different from the eukaryotes, some of the previous researches showed that the existence of poly(A) tails may decrease the stability of mRNA [15,16]. However, the current view of mechanism related to the formation and the regulation of poly(A) tails on the mRNA stability or abundance remains to be smatter [17-19].

In this work, the *sldhAB* gene, which encodes the sorbitol dehydrogenase in *G. oxydans*, was overexpressed with a strong promoter, *P*_*tufB*_ [6]. Inspired by the mechanims from most of the eukaryotes, it is proposed that the mRNA stability could be enhanced by the introduction of extra poly(A) or other similar tails at the 3′-end, such as AAATTT, AAATTTAAA, AAATTTAAATTT, AAATTTAAAAAAA and AAAAAAAAATTT. The mRNA abundance of the *sldhAB* gene with suitable poly(A/T) tails in *G. oxydans* could be significantly enhanced. As a result, both the L-sorbose titer and the stability of the immobilized cells were improved. These results showed that adding some suitable short poly(A/T) tails could significantly improve the mRNA abundance of a specific enzyme, thus facilitate the bioprocesses associated with the enzyme.

## Results and discussion

### qRT-PCR analysis of the *sldhAB* gene

**Figure 1:**
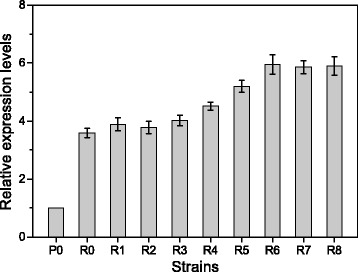
**Transcriptional levels of** ***sldhAB*** **in the recombinant strains.** P0, the control strain; R0, *G. oxydans*-sldhAB; R1, *G. oxydans*-sldhAB1; R2, *G. oxydans*-sldhAB2; R3, *G. oxydans*-sldhAB3; R4, *G. oxydans*-sldhAB4; R5, *G. oxydans*-sldhAB5; R6, *G. oxydans*-sldhAB6; R7, *G. oxydans*-sldhAB7; R8, *G. oxydans*-sldhAB8; the 16S rRNA gene was used as the internal control gene to normalize the results. *Error bars*: Standard deviation (SD) (*n* =3).

In order to identify the role of the poly(A/T) tails in *sldhAB* expression, qRT-PCR analysis was performed with different engineered *G. oxydans* strains grown on D-sorbitol to the beginning of the stationary phase. Expression of the *sldhAB* gene with different tails did not alter the growth phenotype of *G. oxydans* (data not shown). The *sldhAB* expression data obtained were normalized to the expression level in the control strain (Figure 1). *G. oxydans*-sldhAB6, *G. oxydans*-sldhAB7, and *G. oxydans*-sldhAB8 achieved the highest expression levels, which were 6.2-fold and 1.7-fold higher than those of the control and *G. oxydans*-sldhAB without any poly(A/T) tails, respectively. This result showed that the mRNA stability and transcription of the *sldhAB* gene in *G. oxydans* could be markedly enhanced by suitable poly(A/T) tails.

The mRNA level in *G. oxydans* is a dynamic process, which was dominated by both the transcription and mRNA degradation processes. The poly(A) and poly(A/T) tails was located in the downstream of the 3′-UTR and should have minor impact on the expression of mRNA. Therefore, the final mRNA level *in vivo* is actually dominated by the mRNA degradation process. From this point, the improved mRNA level of *sldhAB* should be caused by the decreased RNA degradation process. Thus, this process could be directly reflected from the mRNA level (Figure 1). Compared to eukaryotes, prokaryotes have mRNA of lower stability. Degradation of mRNA in prokaryotes is generally attributed to the combined action of endonucleases and 3′-exonucleases [20,21]. Posttranscriptional mRNA processing includes 5′-capping, splicing, and poly-adenylation at the 3′ end, which contribute to mRNA stability and establish the translational efficiency of mRNA in eukaryotes [22-24]. However, posttranscriptional mRNA processing is not completed independently in prokaryotes. Therefore, an artificial poly(A/T) tail was proposed to slow down the mRNA degradation process in bacteria. As expected, the high *sldhAB* expression levels were achieved when the poly(A/T) tails were linked.

### Effects of poly(A/T) tails on enzyme activity

**Figure 2:**
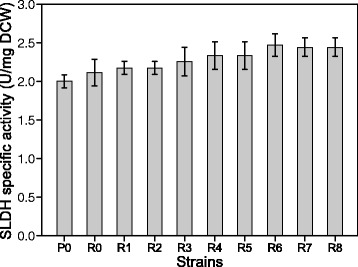
**Effects of poly(A/T) tails on enzyme activity.** P0, the control strain; R0, *G. oxydans*-sldhAB; R1, *G. oxydans*-sldhAB1; R2, *G. oxydans*-sldhAB2; R3, *G. oxydans*-sldhAB3; R4, *G. oxydans*-sldhAB4; R5, *G. oxydans*-sldhAB5; R6, *G. oxydans*-sldhAB6; R7, *G. oxydans*-sldhAB7; R8, *G. oxydans*-sldhAB8. The experiment was performed in 500-mL flasks. *Error bars*: Standard deviation (SD) (*n* =3).

As shown in Figure 2, the specific enzyme activity of SLDH in *G. oxydans* strains was also enhanced when different poly(A/T) tails were linked to the *sldhAB* gene. The highest specific enzyme activity of SLDH was 2.5 U/mg DCW in *G. oxydans* sldhAB6. This result showed that SLDH activity was increased by 5.3% with the *tufB* promoter on the expression vector. Moreover, the existence of poly(A/T) tails further enhanced the activity of SLDH. For example, the SLDH activity in *G. oxydans*-sldhAB6 was improved by 17.1% compared to *G. oxydans*-sldhAB. Besides, some of other novel constitutive strong promoter may improve the abundance of mRNA level of *sldhAB*, thus further improved the enzyme activity of SLDH [25].

As expected, the high SLDH activity levels were achieved when the poly(A/T) tails were linked to the *sldhAB* gene. The enzyme is highly abundant in *G. oxydan-*sldhAB6, so that the proper poly(A/T) tail of the encoding gene led to an increase of the *sldhAB* transcription level when the *sldhAB* gene was overexpressesed. The result indicates that the transcription and expression of the *sldhAB* gene in *G. oxydans* were markedly enhanced by poly(A/T) tails. The results showed that existence of short poly(A/T) tails on mRNA could significantly enhanced the following translational process by enhancing the mRNA stability.

### Effects of poly(A/T) tails on L-sorbose production in shake flasks

**Figure 3:**
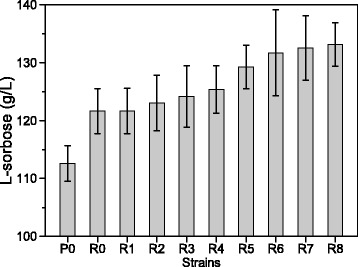
**Effects of poly(A/T) tails on L-sorbose production in shake flasks.** P0, the control strain; R0, *G. oxydans*-sldhAB; R1, *G. oxydans*-sldhAB1; R2, *G. oxydans*-sldhAB2; R3, *G. oxydans*-sldhAB3; R4, *G. oxydans*-sldhAB4; R5, *G. oxydans*-sldhAB5; R6, *G. oxydans*-sldhAB6; R7, *G. oxydans*-sldhAB7; R8, *G. oxydans*-sldhAB8. *Error bars*: Standard deviation (SD) (*n* =3).

Biotransformations of D-sorbitol to L-sorbose by engineered *G. oxydans* strains with the plasmids pBBR-tufB-sldh carrying different poly(A/T) tails were investigated (Figure 3). Overall, the L-sorbose titers from the engineered *G. oxydans* strains were higher than that of the control strain. The L-sorbose titers of *G. oxydans*-sldhAB6, *G. oxydans*-sldhAB7, and *G. oxydans*-sldhAB8 were significantly higher than those of the other engineered strains. A 15.9% increase in L-sorbose titer was obtained for *G. oxydans*-sldhAB6, *G. oxydans*-sldhAB7, and *G. oxydans*-sldhAB8 compared to the control strain. For the three optimal strains, a 9.1% increase in L-sorbose titer was observed when compared to *G. oxydans*-sldhAB. The results showed that enhanced expression of the *sldhAB* gene in *G. oxydans* due to the introduction of poly(A/T) tails improved the production of L-sorbose from D-sorbitol.

### Effects of poly(A/T) tails on L-sorbose production in a 1-L fermenter

**Figure 4:**
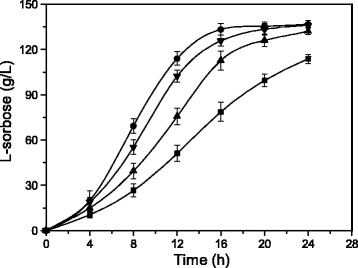
**Effects of poly(A/T) tails on L-sorbose production in a 1-L fermenter.** Solid sqares: wild-type strain, circles: *G. oxydans*-sldhAB6, regular triangles, *G. oxydans*-sldhAB7; inverted triangles: *G. oxydans*-sldhAB8. 0.5 L of initial medium contained 75 g of D-sorbitol, T = 30°C, initial pH = 5.1. *Error bars*: Standard deviation (SD) (*n* = 3).

In order to investigate the effects of poly(A/T) tails on the biotransformation of D-sorbitol and select the best strain from among *G. oxydans*-sldhAB6, *G. oxydans*-sldhAB7, and *G. oxydans*-sldhAB8 for L-sorbose production, batch fermentations of L-sorbose by the optimal strains were conducted in a 1-L fermenter (Figure 4). The L-sorbose titers of the three engineered strains were about 135.0 g/L after 24 h. At 18 h, a maximal L-sorbose titer was achieved by *G. oxydans*-sldhAB6, which was 1.4-fold greater than the original strains. Thus, the culture duration that produces the highest L-sorbose titer in *G. oxydans*-sldhAB6 was shortened by 25.0% compared to the control strain.

### Biotransformation of D-sorbitol to L-sorbose by immobilized *G. oxydans*-sldhAB6

**Figure 5:**
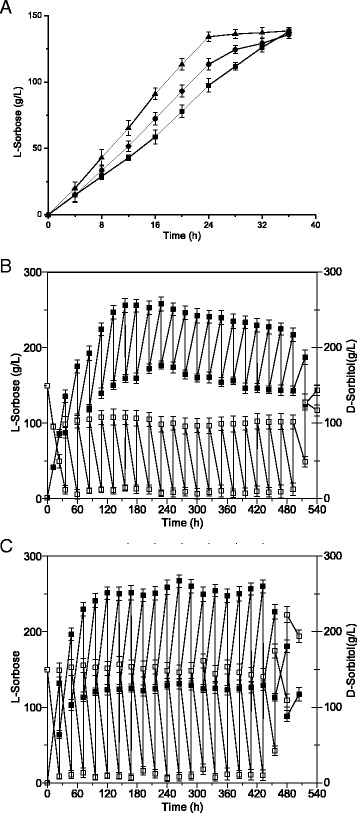
**Effects of immobilization of cells on the conversion of D-sorbitol in a 1-L fermenter. A**. Batch fermentation with immobilized wild-type strains and *G. oxydans*-sldhAB6 cells. Sqares: wild-type strain, circles: *G. oxydans*-sldhAB, triangles: *G. oxydans*-sldhAB6; **B**. Fed-batch fermentation with immobilized wild-type strains. Open squares: D-sorbitol, filled squares: L-sorbose; **C**. Fed-batch fermentation with immobilized *G. oxydans*-sldhAB6 cells. Open squares: D-sorbitol, filled squares: L-sorbose. 0.5 L of initial medium contained 75 g of D-sorbitol, T = 30°C, bead diameter = 2.0 mm, initial pH = 5.1, 2.5% sodium alginate, 0.5% diatomite, 0.25 L of new medium was fed and 0.25 L fermentation broth was discharged every 24 h. *Error bars*: Standard deviation (SD) (*n* = 3).

According to previous researches, immobilized *G. oxydans* cells are ideal for L-sorbose production [26,27]. A similar method that immobilized *G. oxydans*-sldhAB6 cells was adopted in a 1-L fermenter according to our previous work [26]. Overexpression of *sldhAB* gene could enhance the L-sorbose production (Figure 5A). Overexpression of *sldhAB* with suitable poly(A/T) tail showed much better results. The L-sorbose titer for *G. oxydans*-sldhAB6 reached 135.0 g/L after 24 h, which was 1.4-fold of the *G. oxydans* WSH-003. Moreover, the fermentation time of the immobilized *G. oxydans*-sldhAB6 strain was shortened by 33.3% compared to *G. oxydans* WSH-003 and *G. oxydans*-sldhAB, showed that the immobilized *G. oxydans*-sldhAB6 cells were ideal for L-sorbose production. Overexpression of specific gene with strong constitutive promoter may delay the cell growth [27,28]. However, the positive effect of the overexpression was enlarged by immobilization of cells that had already with enhanced D-sorbitol dehydrogenase activity.

Furthermore, to avoid potential delays or inhibition caused by the increased concentration of L-sorbose, an intermittent feeding approach was applied in fed-batch culture. An initial D-sorbitol concentration of 150 g/L was applied. According to the results of Figure 5A, 50 g (*G. oxydans* WSH-003) or 70 g (*G. oxydans*-sldhAB6) of D-sorbitol in a total volume of 0.25 L were fed into the 1-L bioreactor every 24 h. Equal volume of the culture broth was removed before the feeding (Figure 5B,C). The long-term performance of immobilized cells in a 1-L fermenter over a period of 20 days was highly stable. Consequently, the maximal L-sorbose titers were verified for *G. oxydans*-sldhAB6 and *G. oxydans* WSH-003 at 21 days (Figure 5B,C). Both the D-sorbitol consumption and L-sorbose titer decreased sharply after the 20th day. Compared to *G. oxydans* WSH-003, the L-sorbose accumulation for *G. oxydans*-sldhAB6 was improved by 33.7%. The result further demonstrated that the positive effect of the overexpression could be enlarged by immobilization process.

In order to decrease the cost and enhance the production efficiency, oxygen-carriers have been successfully used to enhance the productivity of L-sorbose in batch and fed-batch fermentation [29]. Besides, previous research indicated it was possible to decrease the cost of L-sorbose production by using immobilized *G. oxydans* cells [26,30,31]. However, oxygen-carriers may make downstream processing difficult and the immobilized fermentation requires a longer fermentation period. In this work, enhanced expression of the *sldhAB* gene in *G. oxydans* due to the introduction of poly(A/T) tails improved the production of L-sorbose from D-sorbitol. Besides, the leakage of cells from the beads during repeated usage of immobilized beads should also be monitored to facilitate the downstream process. Compared with the previous strategies, *G. oxydans*-sldhAB6 resulted in a higher productivity without the need for oxygen-carriers. Besides, our method requires a shorter fermentation time than previous research [26,30].

## Conclusions

In summary, poly(A/T) tails were linked to the gene *sldhAB* to enhance mRNA stability and translational efficiency. The results suggested that the mRNA stability and transcription of the *sldhAB* gene in *G. oxydans* were markedly enhanced by poly(A/T) tails. The L-sorbose titer was 33.7% higher in the immobilized *G. oxydans*-sldhAB6 cells. In addition, fed-batch fermentation with immobilized *G. oxydans*-sldhAB6 cells was examined. The long-term performance of immobilized cells in a 1-L fermenter over a period of 20 days showed that the long-term culture process could further amplify the advantage gained by the presence of the poly(A/T) tails. The L-sorbose titer was improved by 33.7% by the immobilization of *G. oxydans*-sldhAB6 cells. The cost of L-sorbose production by *G. oxydans* can be decreased by using this biotransformation process. Therefore, the high activity and stability of immobilized cells make them ideal for large-scale industrial applications.

## Methods

### Strains, plasmids, and growth conditions

*G. oxydans* WSH-003 (CICIM-CU B7004) was obtained from Jiangsu Jiangshan Pharmaceutical Co., Ltd (Jiangsu, China) and stored at −80°C [8]. *G. oxydans* strains were cultivated in D-sorbitol medium (D-sorbitol 150 g/L, yeast extract 6 g/L, CaCO_3_ 2 g/L), at 30°C, 200 rpm [26]. *Escherichia coli* JM109 and *E. coli* pRK2013 were cultivated at 37°C, 200 rpm on Luria-Bertani medium (yeast extract 5 g/L, tryptone 10 g/L, NaCl 10 g/L) with appropriate antibiotics (50 mg/L of kanamycin and/or 100 mg/L ampicillin). All culture media were sterilized at 121°C for 15 min.

### Overexpression of genes in *G. oxydans* WSH-003

Primers are designed with Primer Premier 5 (PREMIER Biosoft International, Palo Alto, CA) based on the bioinformatic analysis of the *G. oxydans* WSH-003 genome sequences [8]. The promoter *tufB* [6] and genes *sldhAB* and *sldhAB* with poly(A/T) tails were PCR-amplified with primer pairs *tufB-*F/*tufB-*R and *sldhAB-*F/*sldhAB-*R, respectively (Table 1). The PCR-amplifed *tufB*, *sldhAB* fragments and *sldhAB* fragments with poly(A/T) tails were digested and inserted into the *Kpn*I/*Xho*I and *Xho*I/*EcoR*I site of the broad host spectrum vector pBBR1MCS-2, respectively, resulting in pBBR-*tufB*, pBBR-*tufBsldhAB* and pBBR-*sldhAB1*, pBBR-*tufBsldhAB2*, pBBR-*tufBsldhAB3*, pBBR-*tufBsldhAB4*, pBBR-*tufBsldhAB5*, pBBR-*tufBsldhAB6*, pBBR-*tufBsldhAB7*, and pBBR-*tufBsldhAB8*. The bases of the poly(A/T) tails of pBBR-*sldhAB1* to pBBR-*sldhAB8* are AAA, AAAAAA, AAAAAAAAA, AAATTT, AAATTTAAA, AAATTTAAATTT, AAATTTAAAAAAA and AAAAAAAAATTT, respectively. Longer poly(A) and poly(A/T) tails, such as AAAAAAAAAAAAAAA, AAATTTAAATTTAAA, AAAAAAAAAAAAAAAAAA, AAATTTAAATTTAAATTT, were also attemp-ted. However, due to the difficulties in chemical synthesis of oligonucleotides or PCR amplification caused by the long iterative A/T bases, none of *sldhAB* gene with poly(A) or poly(A/T) tails longer than 12 or 15 bases could be obtained after repeated attempts. All of the vectors were transformed into *G. oxydans* WSH-003 by triparental mating with the helper strain *E. coli* pRK2013 [32]. The transformants were named as *G. oxydans*-sldhAB and *G. oxydans*-sldhAB1 to *G. oxydans*-sldhAB8, respectively.

**Table 1:** **Primers used in this study**

**Primer**	**Sequnce (5′-3′)**
*tufB*-F	GG**GGTACC**GTACGATGGTAAGAAATCCACTGCC^*^
*tufB*-R	CCG**CTCGAG**CGTCTTTCTCCAAAACCCCGCTCCA
*sldhAB*-F	TTTAACCG**CTCGAG**GTGATTGTGAAATATTTCCAA
*sldhAB*-R	CG**GAATTC**TCAGTGCTTGATGGCATCAG
*sldhAB*-R1	CG**GAATTC**TTTTCAGTGCTTGATGGCATCAG
*sldhAB*-R2	CG**GAATTC**TTTTTTTCAGTGCTTGATGGCATCAG
*sldhAB*-R3	CG**GAATTC**TTTTTTTTTTCAGTGCTTGATGGCATCAG
*sldhAB*-R4	CG**GAATTC**AAATTTTCAGTGCTTGATGGCATCAG
*sldhAB*-R5	CG**GAATTC**TTTAAATTTTCAGTGCTTGATGGCATCAG
*sldhAB*-R6	CG**GAATTC**AAATTTAAATTTTCAGTGCTTGATGGCATCAG
*sldhAB*-R7	CG**GAATTC**TTTTTTAAATTTTCAGTGCTTGATGGCATCAG
*sldhAB*-R8	CG**GAATTC**AAATTTTTTTTTTCAGTGCTTGATGGCATCAG

*Bold: restrict enzyme sites.

### Quantitative real-time PCR (qRT-PCR)

Cells at the beginning of the stationary phase (at 24 h) were harvested at room temperature and immediately frozen in liquid nitrogen. Cells were then stored at −80°C until RNA extraction. Total RNA was extracted with RNAiso™Plus from Takara (Dalian, China). The quantity of total RNA was verified using an Eppendorf Biophotometer (Eppendorf, Hamburg, Germany). The cDNA was synthesized from the total RNA using a PrimeScript RT Reagent Kit (Perfect Real Time) (Takara) according to the manufacturer’s protocol.

Expression levels of different *sldhAB* genes encoding for D-sorbitol dehydrogenase (SLDH) were measured. The 16S rRNA gene was used as the internal standard. Primer pair sets for the genes are listed in Table 2. qRT-PCR analysis was performed in 96-well plates on a LightCycler 480 II instrument (Roche, Mannheim, Germany) using double-stranded-DNA-specific fluorochrome SYBR Green I. Amplification was carried out in a 20-μL (final volume) mixture containing 100 ng of cDNA sample, 0.2 μM forward primer, 0.2 μM reverse primer, and 10 μL of SYBR Premix ExTaq (Takara). A negative control with no cDNA added was systematically included. The amplification procedure involved incubation at 95°C for 40 s for the initial denaturation, followed by 40 cycles consisting of denaturation at 95°C for 5 s, annealing/extension at 55°C for 30 s, and cooling at 50°C for 30 s. The threshold cycle (CT) values were determined with LightCycler software (version 3.3) [33].

**Table 2:** **Oligonucleotides sequence used in qRT-PCR in this study**

**Gene**	**Primers**	**Sequence (5′-3′)**	**Products length (bp)**
16S rRNA	16S rRNA-F	GCGGTTGTTACAGTCAGATG	1478
	16S rRNA-R	GCCTCAGCGTCAGTATCG	
*sldhAB*	*sldh*-F	GCATCAAGTTCCGCAGTG	2670
	*sldh*-R	GTTCCAGTTCGCAATCAGG	

### Measurement of enzyme activity

Cells were harvested at the late-log phase by centrifugation at 9,000 *g* for 10 min. The cells (0.5 g wet weight) were suspended in 1 mL of 50 mM phosphate buffer (pH 7.0) and passed through a French pressure cell press at 20,000 *psi*. After centrifugation to remove intact cells, the supernatant (cell-free extract) was centrifuged at 80,000 *g* for 1 h. The membrane fraction was resuspended into the above buffer (1 mL) [34].

The basal reaction mixture for assaying SLDH activity consisted of 50 mM potassium phosphate buffer (pH 6.0), 0.25 mM DCIP, and 0.325 mM phenazine methosulfate (PMS), which was prepared just before the assay. The cuvette had a 1-cm light path and contained 0.8 mL of the basal reaction mixture, 0.2 mL of 0.4 M D-sorbitol, and the enzyme solution plus water, with a total volume of 1.01 mL. The reference cuvette contained all components except the substrate. The reaction was started at 25°C with D-sorbitol, and the enzyme activity was measured as the initial reduction rate of DCIP at 600 nm. One unit of enzyme activity is defined as the amount of enzyme that catalyzes the reduction of 1 mmol of DCIP per minute [35].

### Measurement of L-sorbose and D-sorbitol

Production of D-sorbitol and L-sorbose was evaluated by HPLC (Agilent 1100 series, Santa Clara, CA), with an Aminex HPX-87H column (300 mm × 7.8 mm; Bio-Rad, Hercules, CA) at 35°C with a flow rate of 0.6 mL/min and 5 mM H_2_SO_4_ as the eluent [36].

### Immobilization of *G. oxydans*

An 18-h culture was harvested during the exponential growth phase and mixed with sodium alginate solution. To prepare the calcium alginate beads, 35 mL of *G. oxydans* sldhAB6 seed culture were added to sodium alginate solution prepared by dissolving 2.5 g of sodium alginate powder to make a 2.5% (w/v) solution in 100 mL of deionized water. The mixture of sodium alginate and seed culture was passed through a peristaltic pump into a 0.15 M CaCl_2_ solution. The beads were uniformly packed and stored in CaCl_2_ solution at 4°C for 3 h and then washed with deionized water to remove residual CaCl_2_ before fermentation [26].

### Statistical analysis

Student’s *t*-test was employed to investigate statistical differences, and samples with *P* <0.05 were considered significant.

## References

[1] Gao LL, Du GC, Zhou JW, Chen J, Liu J (2013). Characterization of a group of pyrroloquinoline quinone-dependent dehydrogenases that are involved in the conversion of L-sorbose to 2-keto-L-gulonic acid in *Ketogulonicigenium vulgare* WSH-001. Biotechnol Progr.

[2] Gao LL, Hu YD, Liu J, Du GC, Zhou JW, Chen J (2014). Stepwise metabolic engineering of *Gluconobacter oxydans* WSH-003 for the direct production of 2-keto-l-gulonic acid from d-sorbitol. Metab Eng.

[3] Zebiri I, Balieu S, Guilleret A, Reynaud R, Haudrechy A (2011). The chemistry of L-Sorbose. Eur J Org Chem.

[4] Macauley-Patrick S, McNeil B, Harvey LM (2005). By-product formation in the d-sorbitol to l-sorbose biotransformation by *Gluconobacter suboxydans* ATCC 621 in batch and continuous cultures. Process Biochem.

[5] Gupta A, Singh VK, Qazi G, Kumar A (2001). *Gluconobacter oxydans*: its biotechnological applications. J Mol Microbiol Biotechnol.

[6] Lu LF, Wei LJ, Zhu K, Wei DZ, Hua Q (2012). Combining metabolic engineering and adaptive evolution to enhance the production of dihydroxyacetone from glycerol by *Gluconobacter oxydans* in a low-cost way. Bioresour Technol.

[7] Kazemi MA, Bamdad H, Papari S, Yaghmaei S (2013). Modeling and control of dissolved oxygen concentration in the fermentation of glucose to gluconic acid. Chem Eng.

[8] Gao LL, Zhou JW, Liu J, Du GC, Chen J (2012). Draft genome sequence of *Gluconobacter oxydans* WSH-003, a strain that is extremely tolerant of saccharides and alditols. J Bacteriol.

[9] Deppenmeier U, Hoffmeister M, Prust C (2002). Biochemistry and biotechnological applications of *Gluconobacter* strains. Appl Microbiol Biotechnol.

[10] Gatgens C, Degner U, Bringer-Meyer S, Herrmann U (2007). Biotransformation of glycerol to dihydroxyacetone by recombinant *Gluconobacter oxydans* DSM 2343. Appl Microbiol Biotechnol.

[11] Zhu K, Lu LF, Wei LJ, Wei DZ, Imanaka T, Hua Q (2011). Modification and evolution of *Gluconobacter oxydans* for enhanced growth and biotransformation capabilities at low glucose concentration. Mol Biotechnol.

[12] Richter JD (1999). Cytoplasmic polyadenylation in development and beyond. Microbiol Mol Biol Rev.

[13] Wu XY, Brewer G (2012). The regulation of mRNA stability in mammalian cells: 2.0. Gene.

[14] Xu F, Cohen SN (1995). RNA degradation in *Escherichia coli* regulated by 3′ adenylation and 5′ phosphorylation. Nature.

[15] Kushner SR (2004). mRNA decay in prokaryotes and eukaryotes: different approaches to a similar problem. IUBMB Life.

[16] Slomovica S, Portnoya V, Liveanua V, Schustera G (2006). RNA polyadenylation in prokaryotes and organelles: Different tails tell different tales. Crit Rev Plant Sci.

[17] Régnier P, Marujo PE, Lapointe J, Brakier-Gingras L (2003). Polyadenylation and Degradation of RNA in Prokaryotes. Translation Mechanisms.

[18] Steege DA (2000). Emerging features of mRNA decay in bacteria. RNA.

[19] Lin H-H, Hsu C-C, Yang C-D, Ju Y-W, Chen Y-P, Tseng C-P (2011). Negative effect of glucose on *ompA* mRNA stability: a potential role of cyclic AMP in the repression of *hfq* in *Escherichia coli*. J Bacteriol.

[20] Arnold TE, Yu J, Belasco JG (1998). mRNA stabilization by the ompA 5′untranslated region: two protective elements hinder distinct pathways for mRNA degradation. RNA.

[21] Rustad TR, Minch KJ, Brabant W, Winkler JK, Reiss DJ, Baliga NS, Sherman DR (2013). Global analysis of mRNA stability in *Mycobacterium tuberculosis*. Nucleic Acids Res.

[22] Gallie DR (1991). The cap and poly (A) tail function synergistically to regulate mRNA translational efficiency. Genes Dev.

[23] Kumar GR, Shum L, Glaunsinger BA (2011). Importin α-mediated nuclear import of cytoplasmic poly (A) binding protein occurs as a direct consequence of cytoplasmic mRNA depletion. Mol Cell Biol.

[24] Perez C, McKinney C, Chulunbaatar U, Mohr I (2011). Translational control of the abundance of cytoplasmic poly (A) binding protein in human cytomegalovirus-infected cells. J Virol.

[25] Shi LL, Li KF, Zhang H, Liu X, Lin JP, Wei DZ (2014). Identification of a novel promoter gHp0169 for gene expression in *Gluconobacter oxydans*. J Biotechnol.

[26] Wang XB, Liu J, Du GC, Zhou JW, Chen J (2013). Efficient production of L-sorbose from D-sorbitol by whole cell immobilization of *Gluconobacter oxydans* WSH-003. Biochem Eng J.

[27] Park YM, Choi ES, Rhee SK (1994). Effect of toluene-permeabilization on oxidation of D-sorbitol to L-sorbose by *Gluconobacter suboxydans* cells immobilized in calcium alginate. Biotechnol Lett.

[28] Goldbeck CP, Jensen HM, TerAvest MA, Beedle N, Appling Y, Hepler M, Cambray G, Mutalik V, Angenent LT, Ajo-Franklin CM (2013). Tuning promoter strengths for improved synthesis and function of electron conduits in *Escherichia coli*. ACS Synth Biol.

[29] Giridhar R, Srivastava A (2000). Productivity enhancement in L-sorbose fermentation using oxygen vector. Enzyme Microb Technol.

[30] Kim HJ, Jong HK, Chul SS (1999). Conversion of D-sorbitol to L-sorbose by *Gluconobacter suboxydans* cells co-immobilized with oxygen-carriers in alginate beads. Process Biochem.

[31] Fidaleo M, Charaniya S, Solheid C, Diel U, Laudon M, Ge H, Scriven L, Flickinger M (2006). A model system for increasing the intensity of whole-cell biocatalysis: Investigation of the rate of oxidation of D-sorbitol to L-sorbose by thin bi-layer latex coatings of non-growing *Gluconobacter oxydans*. Biotechnol Bioeng.

[32] Holscher T, Gorisch H (2006). Knockout and overexpression of pyrroloquinoline quinone biosynthetic genes in *Gluconobacter oxydans* 621H. J Bacteriol.

[33] Yin XX, Madzak C, Du GC, Zhou JW, Chen J (2012). Enhanced α-ketoglutaric acid production in *Yarrowia lipolytica* WSH-Z06 by regulation of the pyruvate carboxylation pathway. Appl Microbiol Biotechnol.

[34] Sugisawa T, Hoshino T (2002). Purification and properties of membrane-bound D-sorbitol dehydrogenase from *Gluconobacter suboxydans* IFO 3255. Biosci Biotechnol Biochem.

[35] Hattori H, Yakushi T, Matsutani M, Moonmangmee D, Toyama H, Adachi O, Matsushita K (2012). High-temperature sorbose fermentation with thermotolerant *Gluconobacter frateurii* CHM43 and its mutant strain adapted to higher temperature. Appl Microbiol Biotechnol.

[36] Zhu YB, Liu J, Du GC, Zhou JW, Chen J (2012). A high throughput method to screen companion bacterium for 2-keto-L-gulonic acid biosynthesis by co-culturing *Ketogulonicigenium vulgare*. Process Biochem.

